# TRPM8-androgen receptor association within lipid rafts promotes prostate cancer cell migration

**DOI:** 10.1038/s41419-019-1891-8

**Published:** 2019-09-09

**Authors:** Guillaume P. Grolez, Dmitri V. Gordiendko, Manon Clarisse, Mehdi Hammadi, Emilie Desruelles, Gaëlle Fromont, Natalia Prevarskaya, Christian Slomianny, Dimitra Gkika

**Affiliations:** 1 Univ. Lille, Inserm, U1003 - PHYCEL - Physiologie Cellulaire, F-59000 Lille, France; 2 Laboratory of Excellence, Ion Channels Science and Therapeutics, Université de Lille, Villeneuve d’Ascq, France; 3 Univ. Lille, CNRS, Central Lille, ISEN, Univ. Valenciennes, UMR 8520, IEMN, F-59000 Lille, France; 4 Univ. de Tours, INSERM, N2C UMR 1069, CHRU de Tours, Department of Pathology, Tours, France

**Keywords:** Urological cancer, Cell migration

## Abstract

In prostate carcinogenesis, androgens are known to control the expression of the transient receptor potential melastatin 8 (TRPM8) protein via activation of androgen receptor (AR). Overexpression and/or activity of TRPM8 channel was shown to suppress prostate cancer (PCa) cell migration. Here we report that at certain concentrations androgens facilitate PCa cell migration. We show that underlying mechanism is inhibition of TRPM8 by activated AR which interacts with the channel within lipid rafts microdomains of the plasma membrane. Thus, our study has identified an additional nongenomic mechanism of the TRPM8 channel regulation by androgens that should be taken into account upon the development of novel therapeutic strategies.

## Introduction

In developed countries, prostate cancer (PCa) is the second most frequently diagnosed cancer and the third most common cause of death by cancer in men^1^. PCa development starts from epithelial cells in the peripheral zone of the prostate and is androgen controlled^2^. Metastasis development at late PCa stages is the main cause of mortality. The main treatment is tumor ablation followed by hormone therapy and androgen suppression in particular. However, survival and proliferation of some PCa cells often become androgen-independent, allowing to escape the above treatment and giving rise to more aggressive forms of cancer^3^. This androgen insensitivity significantly increases PCa mortality rate and suggests that control of PCa progression by androgens have multiple modalities.

The transient receptor potential (TRP) melastatin 8 (TRPM8) channel is involved in PCa, and is one of the most promising clinical targets^4^. Its expression increases during the initial stages of PCa but is reduced after anti-androgen therapy^5^. TRPM8 expression is androgen-dependent with genomic regulation of TRPM8 by the androgen receptor (AR); once activated by androgens, the AR binds to the androgen response elements (AREs) located upstream of the *trpm8* promoter gene^6^. Androgens could also affect the TRPM8 channel in a nongenomic manner^7,8^. However, possible recruitment of the AR to regulation of TRPM8 channel activity and its role in the effects androgens awaits elucidation.

The key event in metastatic progression is cancer cell intravasation that depends on the two processes: cell migration and invasion. Several studies have suggested that TRPM8 plays a central role in the regulation of PCa cell migration and the transition to the androgen-independent aggressive stage of PCa has been shown to positively correlate with loss of TRPM8 expression^9,10^. As the expression and/or activation of TRPM8 suppresses PCa cell migration^10–12^, TRPM8 was pinpointed as potential molecular target antagonizing metastatic transition of PCa.

The aims of this study were to investigate whether variations in androgen levels affect PCa cell migration and, if so, to identify the underlying mechanisms. To this end, we (1) studied the role of the AR in regulation of TRPM8 channel by androgens, (2) analyzed interaction between the AR and TRPM8 proteins and their localization in the plasma membrane (PM) lipid microdomains and (3) assessed the recruitment of this mechanism to control of PCa cell migration.

## Results

### Testosterone inhibits TRPM8 activity

**Fig. 1:**
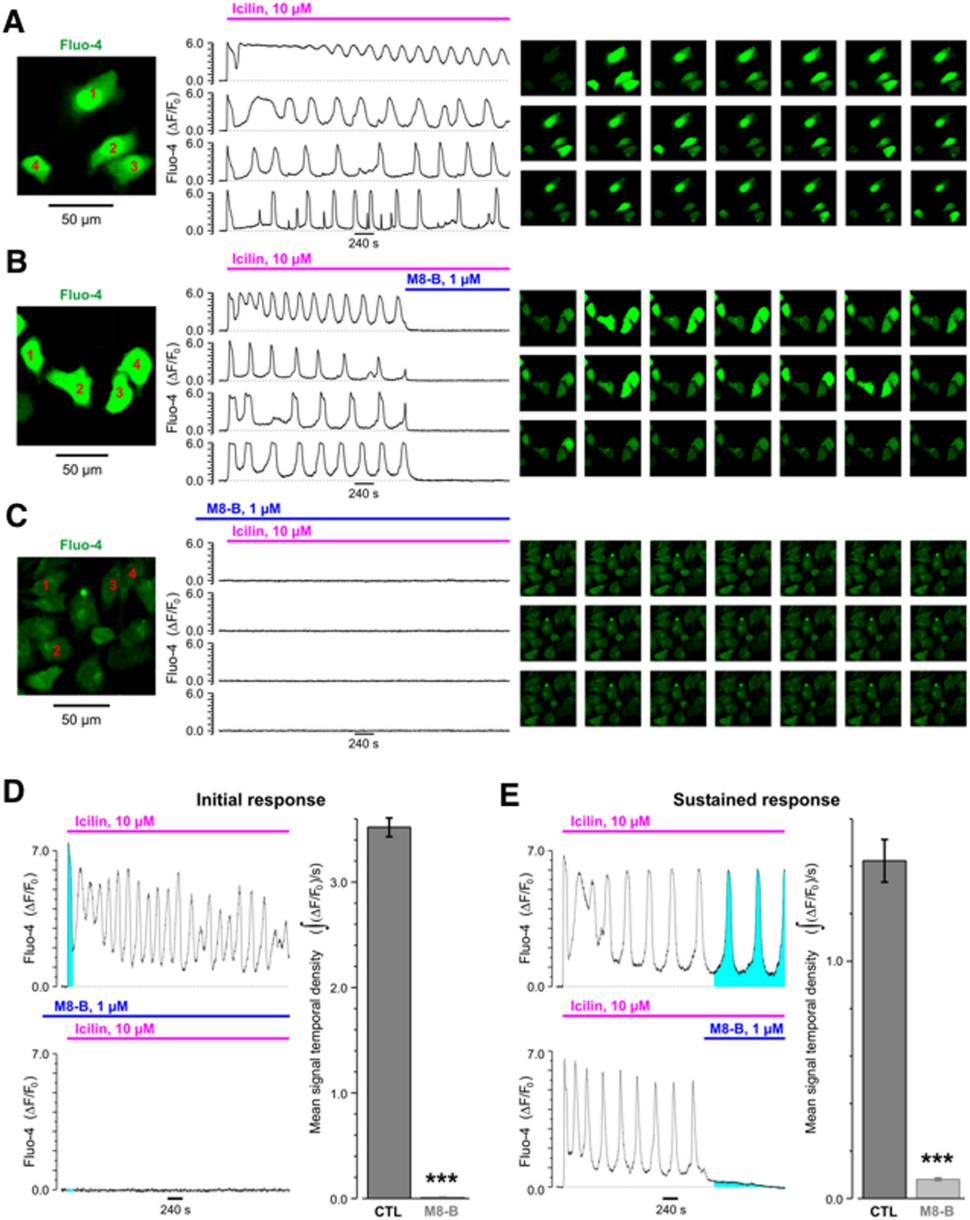
Temporal profile of the [Ca^2+^]_c_ responses induced by 10 µM icilin in PC3 cells transfected with full-length TRPM8 (PC3-M8). Changes in cytosolic Ca^2+^ concentration ([Ca^2+^]_c_), reported by confocal x-y time-series imaging (at 0.5 Hz) of fluo-4 fluorescence, were elicited by stimulation of TRPM8 with 10 µM icilin in PC3-M8 cells. Traces of the relative changes in fluo-4 fluorescence (ΔF/F_0_) in the cells depicted by the numbers (left) are shown from top to bottom, respectively (middle). The galleries (right) show (left to right, top to bottom) every 90^th^ image captured during the imaging protocol. Note that initial [Ca^2+^]_c_ transient is followed by [Ca^2+^]_c_ oscillations persisting for at least 1 h **a**. Also note that block of TRPM8 with selective inhibitor M8-B (1 µM) completely abolished both, the sustained [Ca^2+^]_c_ oscillations **b**, **c** and the initial [Ca^2+^]_c_ transient **c**. The bar diagram plots **d**–**e**, right) compare mean signal temporal densities, calculated as signal mass (left:${\int} {(\Delta } F/F_0)$; cyan: periods of interest) per second for the initial **d** and the sustained **e** response in control (CTL; **d** *n* = 122; **e** *n* = 85) and following TRPM8 inhibition (M8-B; **d** *n* = 72; **e** *n* = 79). ***P < 0.001 (Student t-test)

Prior to an assessment of possible modulation of TRPM8 activity by androgens, we have analyzed temporal pattern of the changes in cytosolic Ca^2+^ concentration ([Ca^2+^]_c_) induced by TRPM8 activation in PCa cells (PC3) overexpressing TRPM8 (PC3-M8). The TRPM8 specificity of the [Ca^2+^]_c_ response was confirmed using several pharmacological agents targeting TRPM8. Using confocal x-y time series imaging of the fluo-4 – loaded cells, we found that stimulation of TRPM8 with 10 µM icilin triggered transient elevation of [Ca^2+^]_c_ followed by [Ca^2+^]_c_ oscillations (Fig. 1a). Interestingly, in the case of stimulation of the same cells with 200 µM menthol, the initial [Ca^2+^]_c_ transient was usually followed by a steady-state plateau-like elevation [Ca^2+^]_c_ while [Ca^2+^]_c_ oscillations were observed only on a few occasions (Fig. S1A). This difference in the temporal pattern of the responses can be attributed to the fact that modes of TRPM8 activation by icilin and menthol differ^13,14^ and/or to effects of menthol not directly linked to TRPM8 activation^15^. Nonetheless, in either case, application of the 1 µM of the selective TRPM8 antagonist, M8-B^16^, completely abolished both, the initial [Ca^2+^]_c_ transient (Fig. 1b, d; Fig. S1B, D) and the sustained response (Fig. 1c, e; Fig. S1C, E). This strongly suggests that both phases of the [Ca^2+^]_c_ response to either icilin or menthol intimately depend on TRPM8 channel activity.

**Fig. 2:**
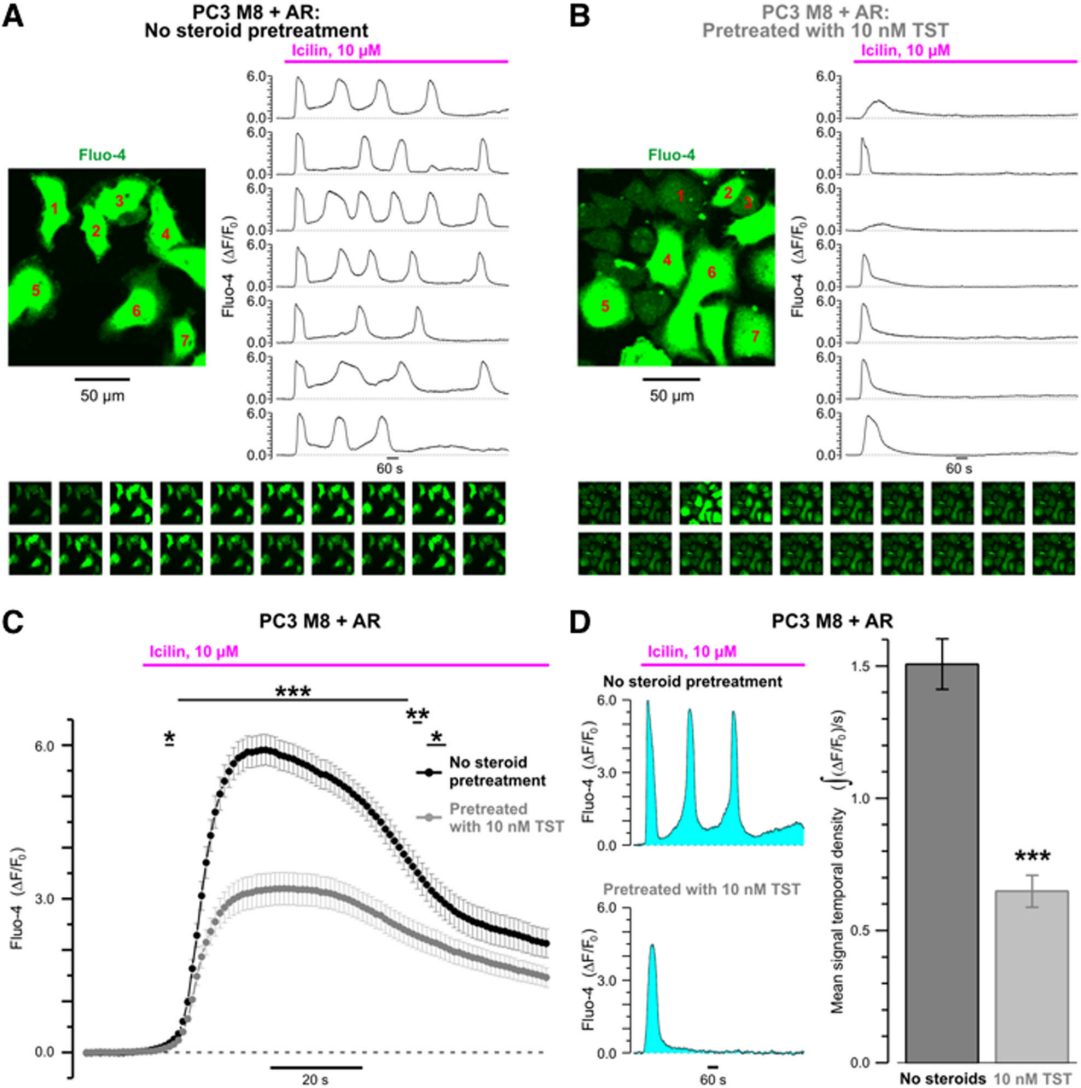
Icilin-induced [Ca^2+^]_c_ responses in PC3 cells co-transfected with androgen receptor (AR) and full-length TRPM8 (PC3-M8-AR) are inhibited by pre-treatment with 10 nM testosterone (TST). Changes in [Ca^2+^]_c_, reported by confocal time-series imaging (at 1 Hz) of fluo-4 fluorescence, were elicited by stimulation of TRPM8 with 10 µM icilin in PC3-M8-AR cells non-treated with steroids **a** or following 15-min incubation with 10 nM TST **b**. The temporal profiles of the relative changes in fluo-4 fluorescence (ΔF/F_0_) in the cells depicted by the numbers (left) are shown from top to bottom, respectively (right). The galleries (bottom) show (left to right, top to bottom) every 60^th^ image captured during the *x*-*y* time series. **c** The plot compares mean ± S.E.M. traces of the initial [Ca^2+^]_c_ transient in non-treated (*n* = 45) and TST-treated (*n* = 44) PC3-M8-AR cells. **d** The bar diagram plot (right) compares corresponding mean signal temporal densities during the entire 30-min records, calculated as signal mass (left: ${\int} {(\Delta } F/F_0)$) per second. ***P < 0.001, **P < 0.01, *P < 0.05 (Student *t*-test)

To assess possible effect of androgens on cytosolic Ca^2+^ mobilization caused by TRPM8 activation we conducted confocal Ca^2+^ imaging on fluo-4 – loaded PC3-M8 overexpressing AR with (Fig. 2) or not (Fig. S2). As demonstrated above, stimulation of TRPM8 with 10 µM icilin triggered transient elevation of cytosolic Ca^2+^ concentration ([Ca^2+^]_c_) followed by [Ca^2+^]_c_ oscillations. This pattern of response did not depend on AR bioavailability and the absence of AR was not generally altered following 15-min pretreatment of the cells (10 nM TST) (Fig. 2a, S2 and Supplemental Movies S1 & S2). However, when TRPM8 was co-expressed with the AR, pretreatment with 10 nM TST significantly attenuated icilin-induced [Ca^2+^]_c_ transient (Fig. 2b, c) and abolished [Ca^2+^]_c_ oscillations (Fig. 2b and Supplemental Movie S2) resulting in significant reduction of the Ca^2+^ signal temporal density (Fig. 1d). These observations indicate that inhibition of TRPM8 by 10 nM TST is mediated via AR activation and that at this concentration TST exerts no direct effect on TRPM8 channel activity.

### Androgen-induced modulation of the PCa cell migration relies on the TRPM8-AR interaction and TRPM8-mediated regulation of [Ca^2+^]_c_

**Fig. 3:**
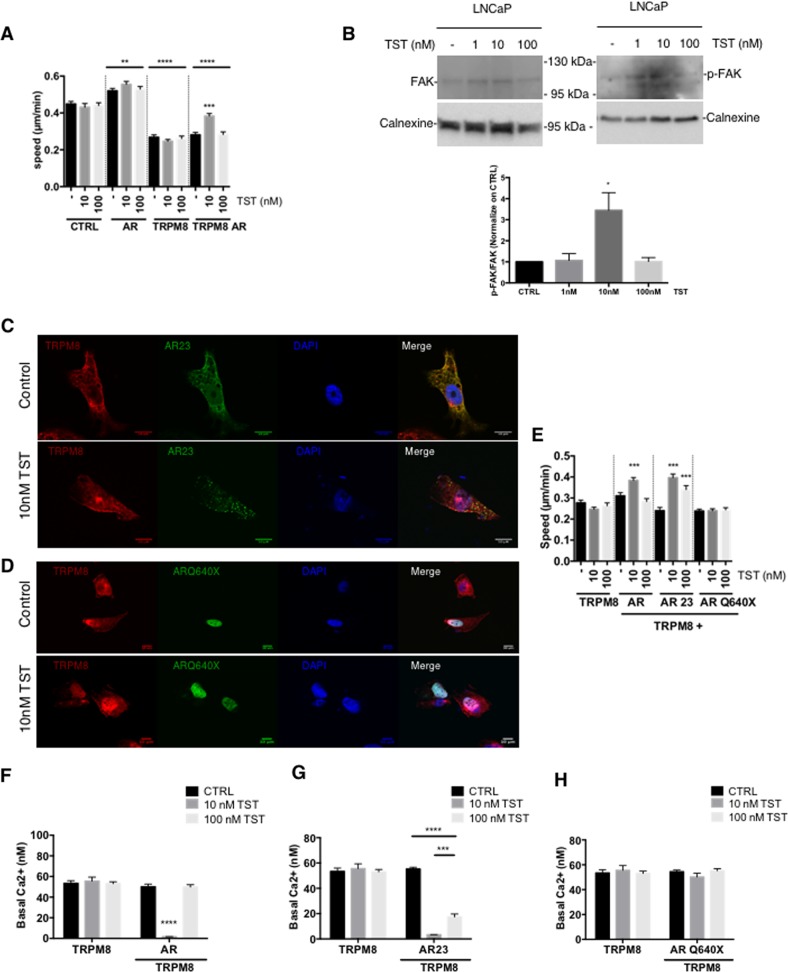
AR modulates PCa cell migration by nongenomc action on TRPM8. **a** PC3 cells (Control, CTRL) were transfected with full-length TRPM8 and/or AR and used in a video microscopy migration assay. The cells were treated with testosterone (TST; at 10 nM or 100 nM) or vehicle only (-) at the beginning of the video microscopy assay. The bar diagram plot compares mean migration speed at different conditions, as indicated. N = 3 independent assays, **P < 0.01, ***P < 0.001, ****P < 0.0001 (Student *t*-test). **b** FAK phosphorylation was analyzed in LNCaP cells treated or not with 1, 10 or 100 nM of TST. Blots are representing of 3 independent assay. Bottom panel: Quantification of FAK phosphorylation was normalized on control condition. *N* = 3 independent assay, *p < 0.05 (Anova). PC3 cells were transfected with full-length TRPM8 and either cytosolic AR mutant (AR23-GFP) (**c**) or nuclear AR mutant (ARQ640X-GFP) **d**, and labeled with anti-TRPM8 antibody. Confocal images were obtained from non-treated cells and those pre-treated with 10 nM TST, as indicated. **e** Bar diagram plot compares mean migration speed in PC3 cells transfected with full-length TRPM8 and/or wtAR, AR23 or ARQ640X, and treated with either TST (at 10 nM or 100 nM) or vehicle only (-) at the beginning of the video microscopy assay. *N* = 3 independent assays, ***P < 0.001, (Student *t*-test). PC3 cells transfected with full-length TRPM8 and/or wtAR (**f**), AR23 (**g**) or ARQ640X (**h**) were loaded with ratiometric Ca^2+^-sensitive indicator Fura-2 and exposed to 0 (CTRL), 10 nM or 100 nM TST for 10 min. The plots compare 340/380 nm fluorescence ratio reflecting basal [Ca^2+^]_c_ at different conditions, as indicated. N = 3 independent assays. ***P < 0.001, ****P < 0.0001. (Student *t*-test)

To investigate the effect of androgens on the PCa cell migration, we performed single cell video tracking using confocal microscopy on PC3 cells overexpressing either AR or TRPM8. Overexpression of either affected the migration speed in TST-independent manner: the former decreased it by 40.19 ± 1.29%, while addition of AR increased it by 15.97 ± 1.29% (Fig. 3a). TRPM8/AR simultaneous overexpression reduced the cell migration speed to the same extend as sole TRPM8 overexpression. However, in contrast to non-transfected cells, exposure of the TRPM8/AR-overexpressing cells to 10 nM TST significantly reversed the TRPM8-dependent inhibition of the cell migration, while exposure to 100 nM still had no effect. These observations suggest that effect of TST is concentration-dependent and that it further requires the expression of both, AR and TRPM8. In PCa cells, it has been shown that FAK activation is critical for focal adhesion formation and, hence, cell migration. We have therefore investigated FAK phosphorylation in LNCaP cells treated or not with 0, 10 and 100 nM of and showed that 10 nM treatment of TST dramatically increased in FAK activation (3.448 ± 0.83 compared to CTRL condition, Fig. 3b).

To further determine whether the observed inhibition of the PCa cell migration was due to a genomic action of the AR that requires nuclear translocation of the receptor, we used the two AR mutants. The first one includes 23 amino acids that preclude AR translocation into the nucleus (Fig. 3b) and, hence, prevent the genomic actions of the receptor (AR23)^17^. The second one includes a stop codon at position 640, leading to constitutive nuclear translocation (Fig. 3c) and activity of the receptor in the absence of TST (ARQ640X)^18^. Interestingly, overexpression of the AR23 showed a similar effect with the wild-type AR on TRPM8-mediated cell migration by inducing a 42.95 ± 1.74% increase in PC3 cell speed upon 10 nM TST application, whereas overexpression of the ARQ640X had no effect on cell migration speed (Fig. 3d). These results suggest that modulation of the PCa migration speed by 10 nM TST is mediated via nongenomic action of the cytosolic AR on TRPM8.

To assess whether the modulation of ongoing TRPM8-mediated Ca^2+^ influx by TST is responsible for its effect on PCa cell migration, we conducted wide-field imaging using ratiometric Ca^2+^ indicator Fura-2. We found that 10 nM, but not 100 nM, TST reduced basal [Ca^2+^]_c_ in the cells co-expressing TRPM8/AR (Fig. 3e). In contrast, the cells co-expressing TRPM8/AR23, showed reduced [Ca^2+^]_c_ following exposure to either 10 nM or 100 nM TST. However the effect of 100 nM TST was significantly weaker (Fig. 3f). More importantly, incubation with neither 10 nM nor 100 nM TST had any effect on [Ca^2+^]_c_ in the cells co-expressing TRPM8/ARQ640X (Fig. 3g).

Altogether, these observations bought us to conclusion that TST accelerates the PCa migration via inhibition of ongoing TRPM8-mediated Ca^2+^ influx by activated AR.

### TRPM8 interacts directly with the AR in a testosterone-dependent manner

**Fig. 4:**
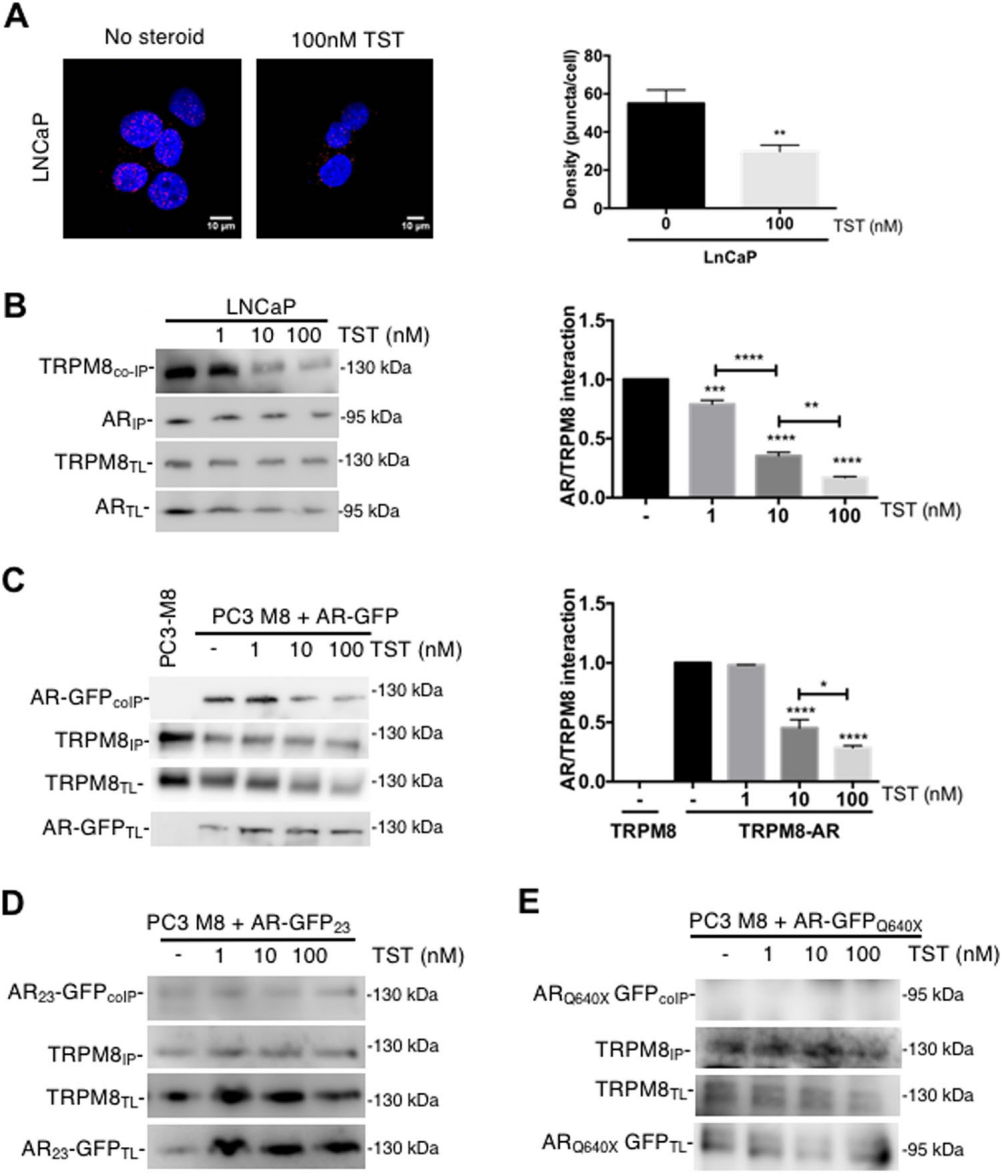
AR-TRPM8 interaction is modulated by AR activation. **a** Left: 3-D images show the results of the AR-TRPM8 proximity ligation assay (PLA) in LNCaP cells non-treated with TST (left) and treated with 100 nM TST (right). Blue: DAPI-stained nuclei; Red: puncta reflecting AR-TRPM8 proximity. Right: bar diagram plot compares the puncta density in non-treated (*n* = 12) and TST-treated (*n* = 19) cells, **P < 0.01, (Student *t*-test). **b** LNCaP cells and **c** PC3 cells transfected with GFP-labeled AR and full-length his-tagged TRPM8 were treated with TST (1, 10 or 100 nM) or vehicle only (-) for 20 min before protein extraction. Total lysates were used for immunoprecipitation against AR **b** or TRPM8 **c** protein. Immunoprecipitation (IP) of the channel was confirmed by immunoblotting for AR or TRPM8 respectively, and the co-immunoprecipitation (coIP) of TRPM8 was detected using an anti-TRPM8 **b** or anti-GFP **c** antibody on the immunoprecipitated or total lysate (TL) fraction. Right panels compare AR-TRPM8 interaction at different conditions, as indicated. **d**, **e** PC3 cells were transfected with the cytoplasmic AR_23_-GFP or nuclear ARQ640X-GFP mutant and full-length his-tagged TRPM8 and immunoprecipitation assay was performed as described in **c**. For each immunoprecipitation assay, *N* = 3 independent experiment. *P < 0.05; **P < 0.01; ***P < 0.005; ****P < 0.0001 (ANOVA)

Proximity ligation assay (PLA) in lymph node carcinoma of prostate (LNCaP) cells demonstrated close proximity of the endogenous TRPM8 and AR, and revealed that their co-localization is reduced (by 50 ± 3.3%) following treatment with 100 nM TST (Fig. 4a). Further analysis of the dependence of the TRPM8-AR interaction in LNCaP cells on TST concentration was performed with co-immunoprecipitation (co-IP) assay. Treatment of the cells with progressively increasing concentrations of TST caused gradual decrease of the TRPM8-AR interaction: by 24.8% at 1 nM, by 64.9% at 10 nM and by 81.7% at 100 nM TST, respectively (Fig. 4b). Similar protocol applied to TRPM8/AR-overexpressing PC3 cells revealed that TRPM8 immunoprecipitated with the AR to the same extent in the absence and in the presence of 1 nM TST, but interaction between the two proteins progressively decreased at the TST concentration range of 10–100 nM: by 58.2%–68.4%, respectively (Fig. 4c). Analysis of the interaction between TRPM8 and AR mutants using co-IP assay demonstrated that TRPM8-AR23 interaction is not affected by TST at concentration range of 1–100 nM (Fig. 4d) and that TRPM8 does not interact at all with ARQ640X (Fig. 4e).

**Fig. 5:**
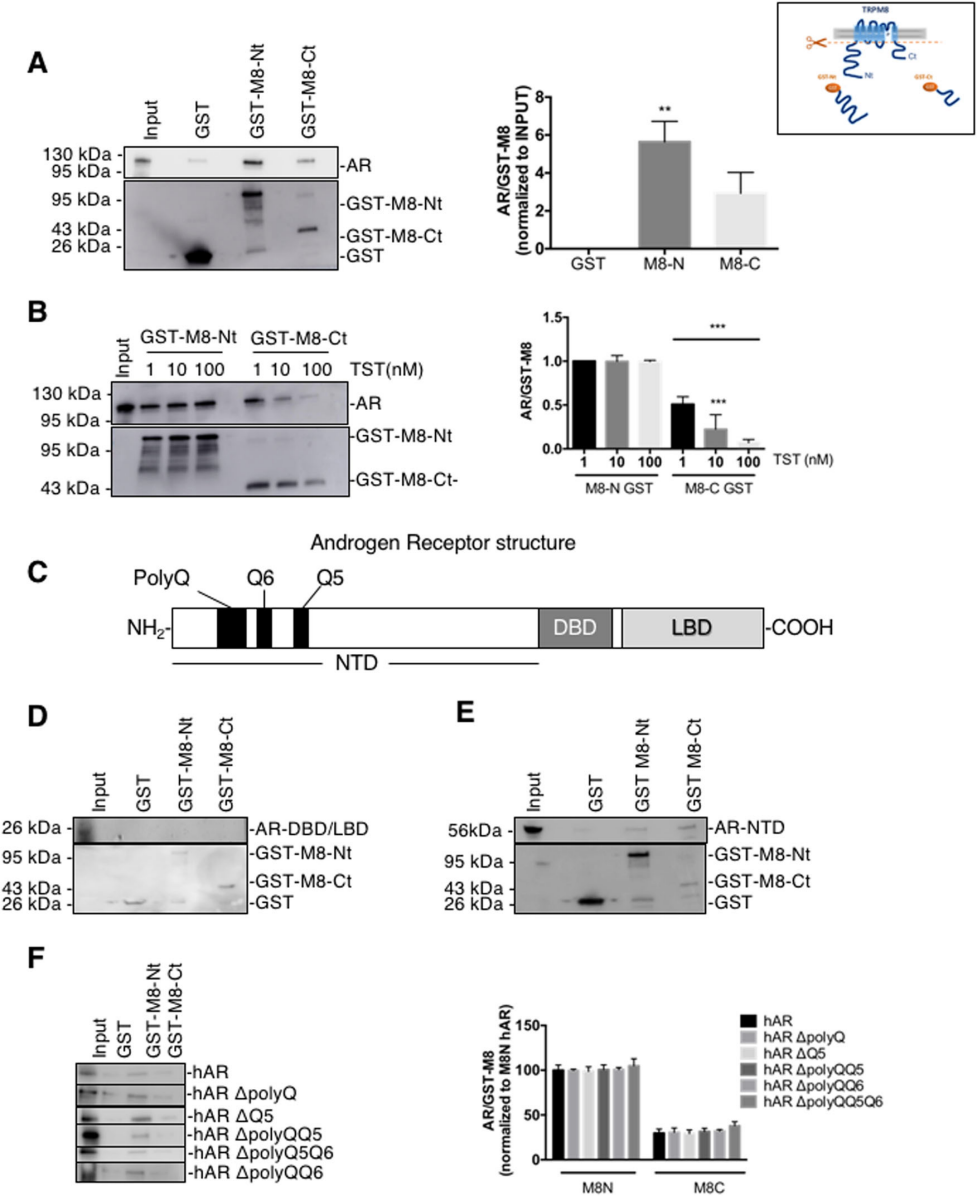
TRPM8 interacts directly with the N-terminal region of AR independently of the polyQ tract. **a** GST pull-down assay of in vitro translated AR and GST, GST fused to the TRPM8 N-terminal tail (GST-Nt) or to C-terminal tail (GST-Ct), as indicated. For the input of the GST pull-down assay, 10% of the in vitro translated AR was used. Right: plot compares the AR-TRPM8 tail interactions normalized to the input. Cartoon (inset) illustrates the GST-Nt and GST-Ct purified fragments. **b** GST pull-down assay of in vitro translated AR and GST, GST fused to GST-Nt or to GST-Ct. In vitro translated AR was treated with 1, 10 or 100 nM testosterone during incubation with the N- or C-terminal tail of TRPM8. Right: plot compares the AR-TRPM8 tail interactions normalized to the input. *N* = 3 independent experiments. **c** Schematic representation of major AR domains: NTD, DBD and LBD. GST pull-down assay of in vitro translated AR-DBD/LBD tails **d** or AR N-terminal tail **e** with GST, GST-Nt or GST-Ct, as indicated. **f** GST pull-down assay of in vitro translated AR mutants harboring polyQ tract deletions (hARΔpolyQ, hARΔQ5, hARΔpolyQQ5, hARΔpolyQQ6 or hARΔpolyQQ5Q6, as indicated) with GST, GST-Nt or GST-Ct. Right: plot compares the interactions between different AR mutants and TRPM8 termini normalized to the input. For each pull-down, *N* = 3 independent experiments.**P < 0.01, ***P < 0.005 (ANOVA)

Further, GST pull-down assay using the GST-tagged N- and C- termini of TRPM8 and in vitro translated AR protein showed that the AR protein interacted with both the N-terminal and C-terminal peptides of TRPM8 (M8-Nt and M8-Ct, respectively) but had 48 ± 7.6% stronger affinity for M8-Nt (Fig. 5a). TST induced dissociation of the TRPM8-AR complex only at the C-terminus of TRPM8. The dose-dependency of this process was characterized by 43 ± 3.8%, 65.6 ± 7.4% and 93.1 ± 1% decreases in the amount of the pulled-down AR by GST-M8-Ct at 1 nM, 10 nM and 100 nM of TST, respectively (Fig. 5b).

To identify the domain of the AR responsible for TRPM8 binding, we generated two AR chimeras: (1) the amino-terminal domain (AR-NTD) of the AR and (2) the fragment of the AR including the central DNA-binding domain (DBD) followed by the ligand-binding domain in the carboxy-terminal end (LBD) responsible for regulation of the transcription by the receptor (AR-DBD/LBD) (Fig. 5c). TRPM8 GST-tagged termini (GST-M8-Nt or GST-M8-Ct) pulled down the in vitro translated AR-NTD (Fig. 5e) but not AR-DBD/LBD (Fig. 5d). The AR-NTD contains three specific sequences composed of poly-glutamine (polyQ) repeats, the length of which varies in humans, and the reduction of which correlates with risk of PCa^19^. We thus evaluated the involvement of the AR NTD polyQ tracts in the TRPM8/AR interaction using several AR mutants characterized by sequential deletions as described in Material and Methods. The pull-down assay with either GST-M8-Nt or GST-M8-Ct revealed no difference between in vitro translated AR polyQ mutant proteins (Fig. 5f).

### The TRPM8-AR interaction occurs within lipid raft microdomains

**Fig. 6:**
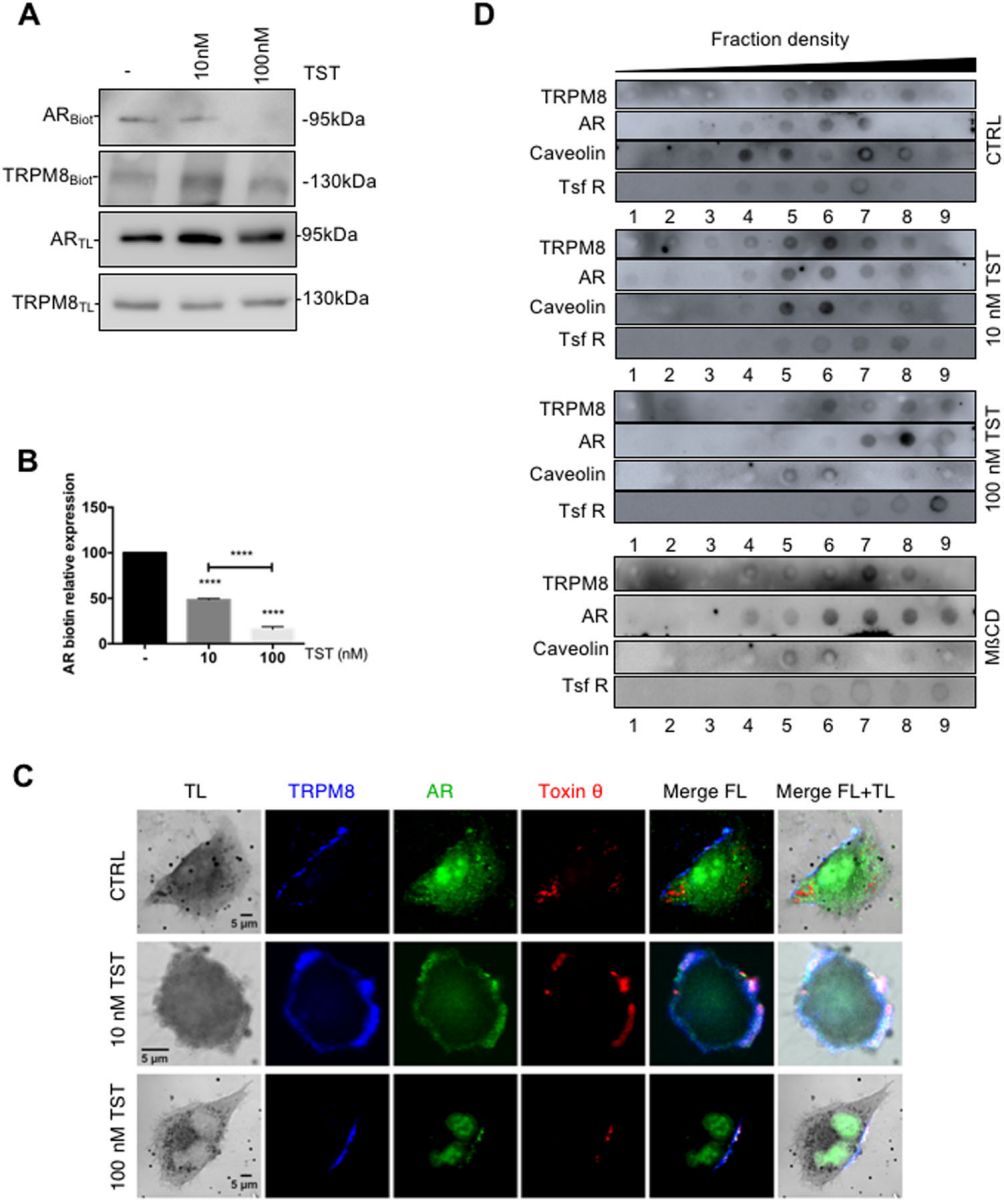
TST-activated AR inhibits TRPM8 in lipid rafts. **a** Expression of TRPM8 and AR in the biotinylated fraction (Biot) and in the total lysate (TL) revealed with immunoblot assay. LNCaP cells were treated with TST (at 10 nM or 100 nM, as indicated) or vehicle only (-) for 15 min before 30-min incubation with 3 mg/ml NHS-LC-LC biotin, and, after lysation, were incubated overnight with neutravidin beads. **b** Plot compares relative AR expression in the plasma membrane (AR_Blot_/AR_TL_) at different TST concentrations. ****P < 0.0001 (Student *t*-test). **c** Confocal images of LNCaP cells treated with anti-TRPM8 (blue) and anti-AR (green) antibodies, and theta toxin-m-cherry (red) labeling cholesterol. Before fixation and staining, the cells were exposed for 15 min to 0 (CTRL), 10 nM or 100 nM TST, as indicater. TL: transmitted light images. **d** Lipid rafts were extracted from LNCaP cells treated with 10 nM, 100 nM TST, vehicle only (-) or 10 mM MßCD. Immunoreactivity against caveolin was used to mark lipid rafts, and immunoreactivity against transferrin receptor (Tsf R) was used as a negative control for lipid rafts. For each assay, *N* = 3 independent experiments were performed

The functional interaction of TRPM8 and AR implies the presence of both proteins in the same cellular microdomain. We first determined the localization of both proteins at the cell surface with biotinylation of LNCaP cells endogenously expressing TRPM8 and AR (Fig. 6a) and PC3 cells overexpressing TRPM8 and AR (Fig. S4A). Immunoblot analysis of the biotinylated fractions of LNCaP cells revealed that expression of the AR in the PM was gradually reduced upon treatment with progressively increasing TST concentrations: by 51.48 ± 1.25% and by 84 ± 2.67% following treatment with 10 nM and 100 nM of TST, respectively (Fig. 6b). Interestingly, in PC3 cells, that do not endogenously express AR, TRPM8/AR co-expression augmented the amount of the AR in the biotinylated fraction by 31.6 ± 6.36% relative to that observed in the cells overexpressing AR solely (Fig. S4A, B). Yet, similarly to LNCaP cells, treatment with TST reduced the plasmalemmal localization of the AR in a dose-dependent manner: decrease by 47.5 ± 2.57% and 91.7 ± 1.83% observed at 10 nM and 100 nM TST, respectively (Fig. S4B). Thus, the results of biotinylation assays demonstrate that TRPM8 promotes plasmalemmal localization of the AR, while TST facilitates its withdrawal from the PM.

We, then, assessed whether functional interaction between TRPM8 and AR takes place in lipid rafts since several studies previously reported that activity of these proteins separately depends on their localization in lipid rafts^20^. We have therefore labeled the cholesterol-rich fractions using the mCherry-tag theta subunit of cholera toxin, toxin θ^21^ and performed the immunofluorescence confocal detection of TRPM8, AR and toxin θ in LNCaP cells (Fig. 6c and Fig. S3; methyl-ß-cyclodextrin (MßCD): negative control). This revealed that co-localization between TRPM8 and the AR in the cholesterol-rich (toxin θ) fractions is much more evident in the cells treated with 10 nM TST than in untreated cells or those exposed to 100 nM TST. Thus, low TST concentrations facilitate translocation and accumulation of the two proteins in the lipid rafts, while at higher TST concentrations these proteins are re-located. Immunoreactivity against caveolin was used to mark lipid rafts, and immunoreactivity against transferrin receptor (TsfR) was used as a negative control for lipid rafts (Fig. 6d). Dot blot analysis revealed that incubation of the cells with 10 nM TST drove both, TRPM8 and AR, proteins to the caveolin-rich/TsfR-poor fractions, while exposure to 100 nM TST induced a retreat of AR from the lipid rafts (fractions 5 and 6, Fig. 6d). The latter is a result of translocation of the AR to the nucleus, as illustrated by confocal imaging (Fig. 6c).

### TRPM8 expression correlates with the expression of the AR in PCa patient tissue samples

**Fig. 7:**
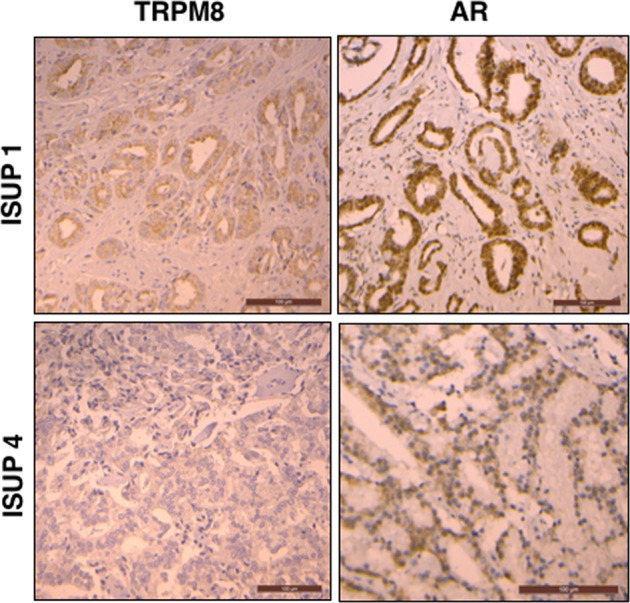
TRPM8 expression is correlated with AR expression in well differentiated PCa. TRPM8 and AR immunoperoxidase staining in the prostate tissue microarray of patient samples. Immunostaining was found in prostate tissues classified as ISUP 1 and corresponding to well differentiated PCa tissue (upper panel), and in the tissues classified as ISUP 4 (lower panel) and corresponding to very low differentiated PCa tissue. The images are representative of 57 samples of ISUP 1 tissue and 143 samples of ISUP 2 to ISUP 4 tissue tested

To explore the clinical relevance of our findings, we examined whether TRPM8 and AR are co-expressed in PCa patient tissue samples. To this end, we analyzed a tissue microarray (TMA) of 200 hormone-naive, clinically localized cancer samples, which included 158 pT2 tumors and 42 pT3 tumors. These samples represented 57 well-differentiated cases (ISUP group 1) and 143 less differentiated cases (ISUP group 2 and higher). In addition, 48 cases of castration-resistant PCa (CRPC) were selected from patients treated exclusively with androgen deprivation therapy. The TMA assay showed TRPM8-positive staining in 36% of the clinically localized, hormone-naive cases and only 4% of CRPC cases (2 cases of 48). Nevertheless, in clinically localized PCa, no correlation was found between TRPM8 expression and progression to the pTNM stage (*P* = 0.2). However, TRPM8 was expressed more frequently in well-differentiated PCa (ISUP group 1) than in less differentiated tumors (ISUP group 2 or higher) (*P* = 0.051). Moreover, positive TRPM8 expression strongly correlated with the AR score (AR score 197 in TRPM8-positive tumors versus 150 in TRPM8-negative tumors) (*P* = 0.002) (Fig. 7).

## Discussion

**Fig. 8:**
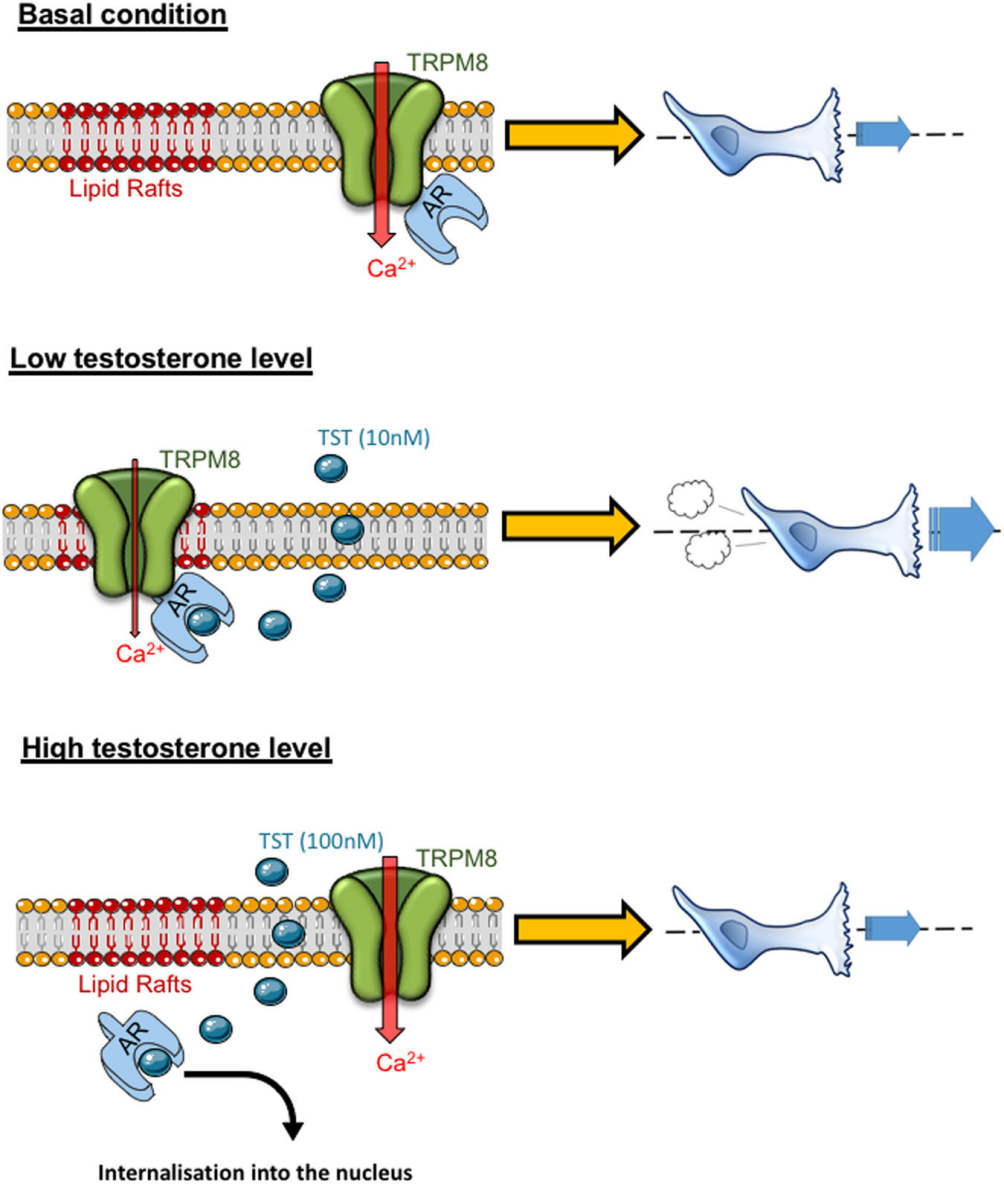
Low testosterone levels inhibit TRPM8 activity by promoting accumulation of the TRPM8-AR complex in lipid rafts. Top panel: in the absence of testosterone, the interaction between TRPM8 and AR takes place in the non-lipid raft domains (non cholesterol-rich domains) of the plasma membrane and does not affect TRPM8 activity and PCa cell migration. Middle panel:exposure of the PCa cells to low testosterone concentration (TST, 10 nM) promotes accumulation of TRPM8-AR complex in lipid rafts, where activated AR inhibits TRPM8 resulting in an increase of PCa cell migration. Bottom panel: exposure of the PCa cells to high testosterone concentration (TST, 100 nM) promotes AR internalization and TRPM8-AR complex dissociation. AR internalization induce a non-lipid rafts membrane localization of TRPM8 that does not affect TRPM8 activity and PCa cell migration

In the present study, we report that androgens directly regulate TRPM8-mediated PCa cell migration. We show that low testosterone (TST) concentrations (10 nM) decrease TRPM8-mediated Ca^2+^ infux, resulting in a significant increase of the cell migration speed. This inhibition of TRPM8 is by direct interaction of the N-terminal domain of the channel with AR forming the protein complex regulated by TST. In total 10 nM TST promotes accumulation of TRPM8 and AR proteins in cholesterol- and caveolin-rich fractions (Fig. 8), while at higher (100 nM) TST concentration this preferential TRPM8/AR localization is lost and the AR is translocated to the nucleus.

Our data show that TRPM8 expression in PC3 reduces cell migration speed in line with previous data^10,11,22^. TRPM8 overexpression without any stimulation is sufficient to induce a decrease in cell migration, suggesting a role of basal TRPM8 activity in this process^22^. Moreover, activation of TRPM8 with icilin, also has been shown to decrease cell migration speed^11,12^. In PCa cells, this inhibition occurs through inactivation of focal adhesion kinase (FAK)^10,22^, a nonreceptor protein tyrosine kinase. FAK phosphorylation is critical for focal adhesion formation and, hence, for cellular processes such as migration or invasion^23^. In line with these observation inactivation of TRPM8 by 10 nM of testosterone increased phosphorylation of FAK with a consequent increase in cell migration. The results of this study provide new dimension to our understanding of the complexity of the processes underlying PCa progression and not only support the view on TRPM8 as a potential molecular target in anti-PCa therapy^9,24^, but also highlight the AR as a TRPM8 regulator protein that should be taken into the account upon development of novel anti-PCa therapeutic strategies.

Indeed, we have demonstrated that in the presence of AR, low doses of TST inhibit TRPM8 basal activity and promote the PCa cell migration (Fig. 3). Other modulators of TRPM8, such as prostate-specific antigen (PSA) or different partner proteins of TRPM8, also affect cell migration^9^. PSA, generally accepted as a PCa marker, is secreted into the fluid of glandular ducts^25^ and acts via bradykinin 2 receptor signaling pathway to facilitate accumulation of the TRPM8 functional channels in the PM^11^. TRP channel-associated factor (TCAF1), not only promotes translocation of the TRPM8 protein to the PM, but also induces an additional open state of the channel, thus, facilitating Ca^2+^ influx into the cell^24^. While regulation of TRPM8 by PSA is mediated via G-protein coupled receptor(s) and downstream signaling cascade, TCAF1 exerts its effects via direct interaction with TRPM8 channel protein, thus, resembling the action of the AR described here.

It was reported previously that androgens may affect TRPM8 in two principal ways: (1) modulation of TRPM8 expression by genomic pathway via AREs upstream of the *trpm8* promoter and (2) modulation of TRPM8 activity by nongenomic pathway via protein-protein interaction and/or TRPM8 phosphorylation^26–28^. Nongenomic regulation of TRP channels by steroid hormones is well appreciated. For example, it was reported that TRPV5 and TRPM6 are regulated by estrogens^29,30^, TRPM3 and TRPV1 are regulated by pregnenolone, and the activity of TRPC3, TRPC4, TRPC5 and TRPC6 is reduced by progesterone^31,32^. Here, using cytosolic (AR23) and nuclear (ARQ640X) AR mutants, we demonstrated that, in similarity to other TRP channels, the TRPM8 channel activity affecting cell migration is regulated via nongenomic pathway. By demonstrating that AR is necessary for the testosterone-mediated TRPM8 regulation affecting [Ca^2+^]_c_ and cell migration, we validate that binding of testosterone to the AR protein occurs near the PM^18^. Indeed biotinylation assays demonstrated localization of AR in accessible vicinity to PM, what, as have been previously demonstrated, is necessary for nongenomic action of androgens in PCa^33,34^.

AR comprises three distinct domains among which polyQ repeats (9 to 36) which are involved in the transactivation of the AR protein by a regulated interaction between the NTD and the C-terminal part of the AR^35^. Variations in the number of the repeats beyond this range were associated with pathology: increased in Kennedy’s disease^36^ and decreased in PCa high risk^19,37^. However, TRPM8 interacts with the N-terminal domain but not within the polyQ tracts of the AR. This pattern of interaction is favored by tetrameric structure of functional TRPM8, where the C- and N-terminal domains of TRPM8 are in close proximity^38^.

The TRPM8 activity can be modulated via its lipid environment since accumulation of TRPM8 in lipid rafts reduces its activity^39^. Here we show further that stimulation with 10 nM TST facilitates accumulation of both, TRPM8 and AR, proteins in the cholesterol-rich fractions, thus promoting AR-TRPM8 interaction and TRPM8 inhibition. TST has molecular structure similar to that of cholesterol (characterized by sterol core) and, hence, like other steroids, may affect the membrane composition, fluidity and interaction between proteins via modulation of their microenvironment^40^. This may partially explain an escape of TRPM8 and AR from the cholesterol-rich fractions observed at 100 nM TST.

The potential mechanism of TRPM8 channels relocation within PM towards lipid rafts may involve the dynamic interaction between TRPM8/AR and possible changes in structural conformation of both proteins. We hypothesize that the strength of TRPM8/AR interaction is a determinant of the two proteins conformation. The conformational changes, in turn, may expose lipid raft localization structure patterns of these proteins, such as putative CRAC (cholesterol recognition amino acid consensus) motif on TRPM8 and caveolin binding domain on AR. Indeed, bioinformatics assay suggested that TRPM8, not unlike TRPV1^41^, comprises a CRAC-like motif ((L/V)-X_1–5_-(Y)-X_1–5_-(K/R))^42^. This CRAC-like motif is located in cytosolic NTD of TRPM8 near its first transmembrane domain and, perhaps, can be engaged upon accumulation of TRPM8 in cholesterol-rich regions of the PM. For example, it was demonstrated that TRPC1 comprises a caveolin1-binding motif facilitating its accumulation in lipid rafts^43^. However, the functional relevance of the TRPM8 CRAC-like motif remains to be elucidated and it would be interesting to investigate in which extend the TRPM8/AR interaction affects or not the recruitment of vesicle-like structures, characteristic of the channel trafficking^44,45^.

Finally, previous studies by Asuthkar et al.^7,8^ suggest direct agonist action of TST on TRPM8, based on lipid bilayers or cell models not expressing AR. TST was applied at picomolar concentrations, which are 10^3^ times lower than physiological levels in men (723.8 ± 221.1 ng/dl = 26.7 ± 8.1 nM)^7,8,46^. In our study, we focused on the role of androgens in PCa progression, we utilized cells expressing both AR and TRPM8 (for which TMA analysis showed to be co-expressed in PCa clinical samples and inversely correlate with PCa aggressiveness) and demonstrated that TST mediates its inhibitory effect on TRPM8 via AR. This inhibitory effect, accelerating the PCa cell migration, is in agreement with the reports of correlation between low serum TST (<230 ng/dl or <8 nM) and tumor aggressiveness, poor prognosis and PCa metastasis^47^.

## Material and methods

### Cell culture

We used human Prostate cancer cells (PC3) with stable TRPM8 overexpression (PC3-TRPM8) and human prostate cancer cells LNCaP. PC3-TRPM8 cells were obtained by a stable transfection of pcdna4-TRPM8 vector. PC3, PC3-TRPM8 and LNCaP cells were grown in RPMI (Invitrogen) supplemented with 10% of fetal bovine serum (Pan Biotech), L-glutamine (5 mM; Sigma-Aldrich) and PenStrep® (100 mg/ml; Sigma-Aldrich).

### Gene silencing and overexpression

Gene overexpression in PC3 and PC3-M8 cells were obtained by lipofection of 1.0 × 10^6^ cells with lipofectamine 3000® (Thermo Fischer Scientific). The constructs used in this study were plasmids carrying the sequences of peGP-AR (addgene), peGFP-AR23 (from Dr Ceraline^17^) and peGFP-ARQ640X (from Dr Ceraline^18^). We transfected 8 µg per dish for the immunoprecipitation assay and 2 µg per well for migration assay.

### Cloning

The coding sequence of the N-terminal tail of TRPM8 (690 aa) and C-terminal tail of TRPM8 was amplified from pGEX6p-2 TRPM8 Nt and pGEX6p-2 TRPM8 Ct vectors^24^. The AR gene, the DNA and ligand binding domain region (AR-DBD/LBD, 366 aa, P556-X920) of AR gene and the N-terminal domain (AR-NTD, 1662 aa, M1-F555) were cloned in the pGEM-T Easy vector (T7 and SP6 RNA polymerase promoter; Promega) from pEGFP-AR vector (addgene #28235) using the following primers: for AR, 5′ ATGGAAGTGCAGTTAGGGCTGGG 3′ and 5′ TCACTGGGTGTGGAAATAGATG 3′; for AR-DBD/LBD, 5′ GCTCTAGAATGCCA CCCCAGAAGACCTGC 3′and 5′ CACTGGGTGTGGAAATAGATG 3′; for AR-NTD, 5′ ATGGAAGTGCAGTTAGGGCTGG 3′ and 5′ TCAGTGGAAAGTAATAGTCAAT GGGCA 3′. AR, AR-DBD/LBD and AR-NTD were both subcloned as EcoRI fragments into the pCMV-TnT vector (CMV promotor; Promega) for in vitro translation.

### Time-lapse video microscopy

Cells were seeded at low density and kept at 37 °C under 5% CO_2_ in an incubator chamber for time-lapse video recording (Okolab). Cell movement was monitored with an inverted microscope (Eclipse Ti-E; Nikon) using a 10×/0.25 NA Plan objective lens.

Images were acquired every 10 min for a time lapse of 10 h with a CCD video camera using NIS-Element software (Nikon). Image stacks were analyzed with ImageJ software and at least 100 cells per condition were manually tracked using the MtrackJ plugin. Dividing cells as well as cells that exited the field of view during the acquisition period were excluded from the analysis of the cell movement speed. Cells were treated with testosterone at different concentration by adding testosterone in growth medium just before the acquisition was commenced. At least six fields for each condition were analyzed in each independent experiment. At least three independent experiments were done for each experimental condition.

### Calcium Imaging

Confocal Ca^2+^ imaging was performed with LSM 510 META confocal workstation using a Plan-Neofluar 40 × 1.3 NA objective (Carl Zeiss, Germany). Fluo-4 was excited by the 488 nm line of 500 mW Argon ion laser (Laser-Fertigung, Hamburg, Germany) and the fluorescence was captured at wavelengths 505–545 nm. Image processing was carried out using LSM 5 software (Zeiss, Oberkochen, Germany) and with custom routines written in IDL (Research Systems, Inc., Boulder, CO, USA). Statistical analysis was performed using MicroCal Origin (MicroCal Software Inc., Northampton, MA, USA).

Basal cytosolic Ca^2+^ concentrations were measured using the ratiometric dye Fura-2/AM (Invitrogen Ltd, UK) and quantified according to Grynkiewicz et al.^48^. Cells were loaded with 2.5 μM of Fura-2/AM (Interchim, France) for 45 min then washed and immersed in the extracellular solution containing 145 mM NaCl, 5 mM KCl, 2 mM CaCl_2_, 1 mM MgCl_2_, 10 mM N-(2-hydroxyethyl)-piperazine-N’-ethanesulfonic acid (HEPES), 10 mM glucose (NaOH to pH 7.35). Observations were performed at 37 °C on an Eclipse Ti microscope using an S Fluor 20×/0.75 NA objective lens (both from Nikon). Images were collected through a Rolera EM-C2 charge-coupled device (CCD) camera (QImaging) controlled with Metafluor software (Molecular Devices). Data were then analyzed with GraphPad Prism 6 software (GraphPad Corporation).

### Proximity ligation assay

Proximity ligation assay was performed using Duolink® In situ red starter kit goat/rabbit (Sigma-Aldrich). LNCaP were seeded at 20 × 10^3^ cells per confocal FluoroDish® (World Precision Instruments). Cells were treated with 100 nM of testosterone or vehicle only for 20 min at 37 °C before fixation and permeabilized with 4% PAF during 10 min. After permeabilization, cells were incubated in the blocking buffer (provided with the kit) for 30 min at 37 °C in a humidified chamber. Cells were incubated with the primary antibodies for 2 h at room temperature: goat anti-TRPM8 antibody (1:200 dilution, Antibodies-online, ABIN572229) and rabbit anti-AR (1:400 dilution; Santa-Cruz, N-20). Spatial distribution of the antibody-labeled proteins was examined using confocal z-sectioning (Zeiss LSM700) and analyzed with Zen 2010 software. The adequacy of the imaging protocol applied to the double-labeled LNCaPs was confirmed by control experiments on the single-labeled cells. Each experiment was repeated at least 3 times.

### Western blot analyses

Cells were seeded in Petri dishes with the appropriate medium and grown to a confluency of 80%. Before cell lysis, Petri dishes were kept on ice and the cells were washed twice with ice-cold PBS. Cells were lysated in RIPA Buffer (25 mM Tris-HCl (pH 7.6), 150 mM NaCl, 1% NP-40, 1% sodium deoxycholate, 0.1% SDS) supplemented with the following protease inhibitors: 2 mg/ml aprotinin, 1 mM Na orthovanadate, 0.1 mM PMSF and 10 mM Sodium Fluoride. Lysates were centrifuged at 4 °C for 10 min at 12,000 × *g*. Protein concentrations were determined using a Bicinchoninic Acid Kit (BCA kit, Sigma). 50 µg of sample were resuspended in SDS sample buffer, heated at 95 °C for 5 min and separated on 10% pre-cast SDS gel (Biorad). Polyvinylidene fluoride membranes were properly blocked and then incubated overnight with rabbit anti-TRPM8 (1:400 dilution; Abcam, ab109308) and with rabbit anti-AR (1:400 dilution; Santa-Cruz, N-20). The membrane was then washed with TBS containing 0.1% Tween 20 and incubated with the appropriate horseradish-peroxidase-conjugated antibodies (SantaCruz). Chemiluminescence assays were conducted using the SuperSignal West Dura chemiluminescent substrate (Thermo Fischer Scientific).

### Immunoprecipitation assay

PC3-TRPM8 cells were transfected with peGFP-AR (wild-type, 23 or Q640X). After 48 h of transfection, the cells were incubated with different concentration of testosterone (1, 10 or 100 nM) during 20 min at 37 °C and washed twice with PBS. Cells were incubated on ice in lysis buffer (1% Triton X-100, 1% sodium deoxycholate, 150 mM NaCl, 10 mM NaKPO_4_, pH 7.2, supplemented with anti-protease cocktail; Sigma-Aldrich). After lysate centrifugation (12,000 × *g* for 10 min at 4 °C), the protein concentration was determined using BCA assay (Thermo Fischer Scientific). An equal amount of supernatants was incubated overnight at 4 °C with 40 µl of His-tag beads (dynadeads® His-tag, Thermo Fischer Scientific) in IP buffer (20 mM NaH_2_PO_4_, 150 mM NaCl, pH 8). The pellet was washed three times in IP buffer, resuspended in SDS sample buffer, heated at 95 °C for 5 min and separated on 10% pre-cast SDS–PAGE gels (Biorad). SDS-PAGE gels were analyzed by immunoblotting using rabbit anti-androgen receptor (AR) (1:400 dilution; Santa-Cruz, N-20) and rabbit anti-TRPM8 (1:400 dilution; Abcam, ab109308) antibodies. Each experiment was repeated at least three times. TRPM8 antibody specificity was validated using siRNA against the TRPM8 channel (Fig. S4D) as described previously^24^.

### GST-fusion proteins and pull-down assay

TRPM8 N- and C-terminal tail GST-fusion proteins were produced and purified as described previously^24^. The involvement of the AR NTD polyQ tracts in the TRPM8/AR interaction was evaluated using several AR mutants characterized by sequential deletions: (1) deletion of the longest tract (hAR ΔpolyQ), (2) deletion of the tract of 5 glutamine repetitions (hAR ΔQ5), (3) simultaneous deletion of the longest tract and the one with 6 glutamine repetitions (hAR ΔpolyQQ6), (4) simultaneous deletion of the longest tract and the one with 5 glutamine repetitions (hAR ΔpolyQQ5) and (5) simultaneous deletion of all three tracts (hAR ΔpolyQQ5Q6) 20. For the direct interaction assay, PCMV-TNT AR, PCMV-TNT DBD/LBD or PCMV-TNT NBD vector were translated in vitro using the TNT Quick Coupled Transcription/Translation Systems kit (Promega) and the FluoroTect^TM^ GreenLys in vitro Translation Labeling System (Promega). In vitro translated proteins were incubated overnight at 4 °C together with the purified GST-fusion proteins in the presence or absence of testosterone. Subsequently, beads were washed extensively and bound proteins were eluted with SDS-PAGE loading buffer, separated on 10% SDS-PAGE gels and visualized by fluorescence imaging or immunoblotting using anti-AR (1:400 dilution; Santa-Cruz, N-20) and anti-GST (1:1000 dilution) antibodies (Bio-Imager Amersham Imager 600, GE, Healthcare, France). Each experiment was repeated at least three times.

### Biotinylation assay

The biotinylation assay was performed using the NHS-LC-LC-biotin kit (Pierce, Etten-Leur, The Netherlands) after homogenizing the cells in 600 µl of lysis buffer, as described previously^24^. Biotinylated proteins were precipitated using neutravidin-agarose beads (Pierce). TRPM8 and AR expression was analyzed by immunoblotting of the precipitates (PM fraction) or of total cell lysates using the anti-TRPM8 (1:400 dilution; Abcam, ab109308) and the anti-AR antibodies (1:400 dilution; Santa-Cruz, N-20). Each experiment was repeated at least three times.

### Labeling of cholesterol rich fractions

Cholesterol was labeled with the theta subunit of cholera toxin provided by Dr. Donatienne Tyteca. Theta toxin was produced in bacteria with the pET28/his-mCherry-theta-D4 vector, as previously described^49^ and purified by Ni-Nta affinity column. Cells were incubated for 10 min with 1.25 µM of theta toxin at room temperature in 0% SVF medium supplemented with 1.25 µM of fatty acid-free BSA (Sigma-Aldrich) and cleared from aggregates by centrifugation at 14,000 × *g* for 5 min. Following cholesterol labeling, the cells were washed 4 times with 0% SVF medium and kept on 0 steroid medium for confocal imaging (Zeiss LSM780).

### Lipids raft extraction

Lipid rafts from LNCaP cells or TRPM8/AR overexpressing PC3 cells were obtained, as described previously^50^. Briefly, cells were homogenized in lysis buffer A (20 mM HEPES, 5 mM EDTA, 150 mM NaCl, pH 7.4) supplemented with 0.5% Triton X-100 mixed with complete protease inhibitors (Roche Applied Science) and solubilized by agitation during 30 min at 4 °C. The lysates were passed 10 times through a 21-gauge needle, and 500 µl of lysate were mixed with 700 µl of a 60 % OptiPrep^TM^ solution (Axis-Shield, Oslo, Norway) and applied to the bottom of a centrifugation tube. A discontinuous OptiPrep^TM^ gradient was prepared by diluting 10 ml of 30% OptiPrep^TM^ in 0.5% Triton X-100 containing lysis buffer A and 1 ml of buffer alone on the top. Gradients were centrifuged at 178,000 × *g* for 4 h at 4 °C in a Beckman Optima XPN-80 ultracentrifuge using a SW40-Ti rotor. After centrifugation, nine fractions (1.3 ml each) were collected from the top, and equal volumes of each fraction were analyzed by dot blot.

### Patient samples

Hormone naive clinically localized cancer samples (HNCLC) (*n* = 200) were obtained from patients treated with radical prostatectomy for localized PCa, including 158 pT2 tumors and 42 pT3. Fifty-seven PCa were well differentiated (ISUP group 1), and 143 less differentiated (ISUP group 2 and more).

Forty-eight cases of castration resistant prostate cancers (CRPC) were selected from patients treated exclusively with androgen deprivation therapy (ADT). Tissues were collected by transurethral resection, performed due to the lower urinary tract symptoms associated with local tumor progression.

Written informed consents were obtained from patients in accordance with the requirements of the medical ethic committee, Comité de Protection des Personnes of Tours Universitary Hospital of the 18th of February (DC-2014-2045).

### Immunohistochemistry on tissue micro-array (TMA)

#### TMA Construction

TMAs were constructed using formalin-fixed paraffin-embedded tissue samples. Original slides stained with hematoxylin-eosin were reviewed using the 2017 TNM classification and the 2014 modified “Gleason” system. For each case, 3 cores 0.6 diameter were transferred from the selected areas to the recipient block, using a TMA workstation (Manual Tissue Arrayer MTA Booster, Alphelys, France). Serial 3 µm sections of the TMA blocks were used for immunohistochemistry. One section out of ten was stained with hematoxylin-eosin to check that the cores adequately represented diagnostic areas.

#### Immunohistochemistry

Slides were deparaffinized, rehydrated, and heated in citrate buffer pH 6 for antigenic retrieval. After block of endogenous peroxidase with 3% hydrogen peroxide, the slides were exposed to the primary antibodies: anti-TRPM8 (Antibodies Online, 1:300 dilution, 1 h incubation) and anti-AR (Abnova, 1:500 dilution, 1 h incubation). Immunohistochemistry was performed using the streptavidin-biotin-peroxydase method with diaminobenzidine as the chromogen (Kit LSAB, Dakocytomation, Glostrup, Denmark). Slides were finally counterstained with haematoxylin. Negative controls were performed by either omission of the primary antibody from staining protocol or by incubation with an irrelevant antibody.

#### Scoring of antibody staining

Staining for TRPM8 was scored as “–“ (staining not detected) and “+” (staining detected). AR-positive cells were expressed as a percentage of total epithelial cells. Moreover, intensity of AR staining was also scored as moderate “1” or high “2”. AR expression level was then defined as the product of percentage and intensity (range: 0–200).

### Statistical analysis

Values are expressed as means ± SEM. The statistical significance of differences between groups was determined by analysis of variance (ANOVA) followed by pairwise comparison using Tukey’s method for migration assays, immunoprecipitation or GST-pull down quantification. Student t-test was used for calcium imaging, PLA and biotinylation assays. The threshold for statistical significance was set at the 0.05 level. Statistical analysis was performed with GraphPad Prism 6 software (GraphPad Corporation) or, in the case of TMA, with StatView 5.0 software (Abacus Concepts, Berkeley, CA). Comparison between groups was performed using the χ2 test for categorical data and nonparametric Mann-Whitney *U* test for continuous data.

## Supplementary information

Supplemental Movie S1

Supplemental Movie S2

Supplemental Figures 1 to 4 and their legends

## References

[1] Ferlay J (2015). Cancer incidence and mortality worldwide: sources, methods and major patterns in GLOBOCAN 2012. Int. J. Cancer.

[2] Cunha GR (1987). The endocrinology and developmental biology of the prostate. Endocr. Rev..

[3] Bubendorf L (2000). Metastatic patterns of prostate cancer: an autopsy study of 1,589 patients. Hum. Pathol..

[4] Tsavaler L, Shapero MH, Morkowski S, Laus R (2001). Trp-p8, a novel prostate-specific gene, is up-regulated in prostate cancer and other malignancies and shares high homology with transient receptor potential calcium channel proteins. Cancer Res..

[5] Henshall SM (2003). Survival analysis of genome-wide gene expression profiles of prostate cancers identifies new prognostic targets of disease relapse. Cancer Res..

[6] Bidaux G (2005). Evidence for specific TRPM8 expression in human prostate secretory epithelial cells: functional androgen receptor requirement. Endocr. Relat. Cancer.

[7] Asuthkar S (2015). The TRPM8 protein is a testosterone receptor: I. Biochemical evidence for direct TRPM8-testosterone interactions. J. Biol. Chem..

[8] Asuthkar S, Velpula KK, Elustondo PA, Demirkhanyan L, Zakharian E (2015). TRPM8 channel as a novel molecular target in androgen- regulated prostate cancer cells. Oncotarget.

[9] Grolez GP, Gkika D (2016). TRPM8 puts the chill on prostate cancer. Pharmaceuticals.

[10] Zhu G (2011). Effects of TRPM8 on the proliferation and angiogenesis of prostate cancer PC-3 cells in vivo. Oncol. Lett..

[11] Gkika D, Flourakis M, Lemonnier L, Prevarskaya N (2010). PSA reduces prostate cancer cell motility by stimulating TRPM8 activity and plasma membrane expression. Oncogene.

[12] Genova T (2017). TRPM8 inhibits endothelial cell migration via a non-channel function by trapping the small GTPase Rap1. J. Cell Biol..

[13] Bidaux G (2012). Regulation of activity of transient receptor potential melastatin 8 (TRPM8) channel by its short isoforms. J. Biol. Chem..

[14] Bharate SS, Bharate SB (2012). Modulation of Thermoreceptor TRPM8 by Cooling Compounds. ACS Chem. Neurosci..

[15] Farco JA, Grundmann O (2013). Menthol-pharmacology of an important naturally medicinal ‘cool’. Mini Rev. Med Chem..

[16] Almeida MC (2012). Pharmacological blockade of the cold receptor TRPM8 attenuates autonomic and behavioral cold defenses and decreases deep body temperature. J. Neurosci..

[17] Jagla M (2007). A splicing variant of the androgen receptor detected in a metastatic prostate cancer exhibits exclusively cytoplasmic actions. Endocrinology.

[18] Lapouge G (2007). Unexpected paracrine action of prostate cancer cells harboring a new class of androgen receptor mutation—A new paradigm for cooperation among prostate tumor cells. Int. J. Cancer.

[19] Callewaert L (2003). Implications of a polyglutamine tract in the function of the human androgen receptor. Biochem. Biophys. Res. Commun..

[20] Freeman MR, Cinar B, Lu ML (2005). Membrane rafts as potential sites of nongenomic hormonal signaling in prostate cancer. Trends Endocrinol. Metab..

[21] Mound A (2017). Non-senescent keratinocytes organize in plasma membrane submicrometric lipid domains enriched in sphingomyelin and involved in re-epithelialization. Biochim. Biophys. Acta.

[22] Yang Z-H, Wang X-H, Wang H-P, Hu L-Q (2009). Effects of TRPM8 on the proliferation and motility of prostate cancer PC-3 cells. Asian J. Androl..

[23] Schlaepfer DD, Mitra SK, Ilic D (2004). Control of motile and invasive cell phenotypes by focal adhesion kinase. Biochim. Biophys. Acta.

[24] Gkika D (2015). TRP channel-associated factors are a novel protein family that regulates TRPM8 trafficking and activity. J. Cell Biol..

[25] Stenman U-H, Leinonen J, Zhang W-M, Finne P (1999). Prostate-specific antigen. Semin. Cancer Biol..

[26] Bonaccorsi L (2008). Prostate cancer: a model of integration of genomic and non-genomic effects of the androgen receptor in cell lines model. Steroids.

[27] Foradori CD, Weiser MJ, Handa RJ (2008). Non-genomic actions of androgens. Front. Neuroendocrinol..

[28] Heinlein CA, Chang C (2002). The roles of androgen receptors and androgen-binding proteins in nongenomic androgen actions. Mol. Endocrinol..

[29] Cao G (2009). Regulation of the epithelial Mg2+ channel TRPM6 by estrogen and the associated repressor protein of estrogen receptor activity (REA). J. Biol. Chem..

[30] Chen S-C, Wu F-S (2004). Mechanism underlying inhibition of the capsaicin receptor-mediated current by pregnenolone sulfate in rat dorsal root ganglion neurons. Brain Res..

[31] Majeed Y (2012). Pregnenolone sulphate-independent inhibition of TRPM3 channels by progesterone. Cell Calcium.

[32] Miehe S (2012). Inhibition of diacylglycerol–sensitive TRPC channels by synthetic and natural steroids. PLoS ONE.

[33] Papadopoulou N, Charalampopoulos I, Alevizopoulos K, Gravanis A, Stournaras C (2008). Rho/ROCK/actin signaling regulates membrane androgen receptor induced apoptosis in prostate cancer cells. Exp. Cell Res..

[34] Papakonstanti EA, Kampa M, Castanas E, Stournaras C (2003). A rapid, nongenomic, signaling pathway regulates the actin reorganization induced by activation of membrane testosterone receptors. Mol. Endocrinol..

[35] Tan MHE, Li J, Xu HE, Melcher K, Yong E (2015). Androgen receptor: structure, role in prostate cancer and drug discovery. Acta pharmacologica Sin..

[36] LaFevre-Bernt MA, Ellerby LM (2003). Kennedy's disease. Phosphorylation of the polyglutamine-expanded form of androgen receptor regulates its cleavage by caspase-3 and enhances cell death. J. Biol. Chem..

[37] Giovannucci E (1997). The CAG repeat within the androgen receptor gene and its relationship to prostate cancer. Proc. Natl Acad. Sci. USA.

[38] Yin Y (2018). Structure of the cold- and menthol-sensing ion channel TRPM8. Science.

[39] Morenilla-Palao C, Pertusa M, Meseguer V, Cabedo H, Viana F (2009). Lipid Raft Segregation Modulates TRPM8 Channel Activity. J. Biol. Chem..

[40] Whiting KP, Restall CJ, Brain PF (2000). Steroid hormone-induced effects on membrane fluidity and their potential roles in non-genomic mechanisms. Life Sci..

[41] Morales-Lázaro Sara L., Lemus Luis, Rosenbaum Tamara (2016). Regulation of thermoTRPs by lipids. Temperature.

[42] Fantini J, Barrantes FJ (2013). How cholesterol interacts with membrane proteins: an exploration of cholesterol-binding sites including CRAC, CARC, and tilted domains. Front. Physiol..

[43] Lockwich TP (2000). Assembly of Trp1 in a signaling complex associated with caveolin-scaffolding lipid raft domains. J. Biol. Chem..

[44] Toro CA (2015). Agonist-Dependent Modulation of Cell Surface Expression of the Cold Receptor TRPM8. J. Neurosci..

[45] Ghosh D (2016). VAMP7 regulates constitutive membrane incorporation of the cold-activated channel TRPM8. Nat. Commun..

[46] Bhasin S (2011). Reference ranges for testosterone in men generated using liquid chromatography tandem mass spectrometry in a community-based sample of healthy Nonobese young men in the Framingham heart study and applied to three geographically distinct cohorts. J. Clin. Endocrinol. Metab..

[47] Tu H (2017). Low serum testosterone is associated with tumor aggressiveness and poor prognosis in prostate cancer. Oncol. Lett..

[48] Grynkiewicz G, Poenie M, Tsien RY (1985). A new generation of Ca^2+^ indicators with greatly improved fluorescence properties. J. Biol. Chem..

[49] Carquin M (2014). Endogenous sphingomyelin segregates into submicrometric domains in the living erythrocyte membrane. J. Lipid Res..

[50] Valero M, Morenilla-Palao C, Belmonte C, Viana F (2010). Pharmacological and functional properties of TRPM8 channels in prostate tumor cells. Pflügers Arch. Eur. J. Physiol..

