# Supplementary material for: TRPM8-androgen receptor association within lipid rafts promotes prostate cancer cell migration
Source: Cell Death Dis. 2019 Sep 9;10(9):652. doi: 10.1038/s41419-019-1891-8 (PMC6733924; doi:10.1038/s41419-019-1891-8)
Supplement: Supplementary file 3 — Supplemental Figures 1 to 4 and their legends [file 41419_2019_1891_MOESM3_ESM.pdf]

Supplemental materiel of the Manuscript CDDIS-19-0794

TRPM8-Androgen Receptor Association within Lipid Rafts Promotes Prostate Cancer Cell Migration

Guillaume P. Grolez[1,2,#], Dmitri V. Gordiendko[1,2,#], Manon Clarisse[1,2], Mehdi Hammadi[3], Emilie Desruelles[1,2], Gaëlle Fromont[4], Natalia Prevarskaya[1,2], Christian Slomianny[1,2] & Dimitra Gkika[1,2,*]

Supplemental S1

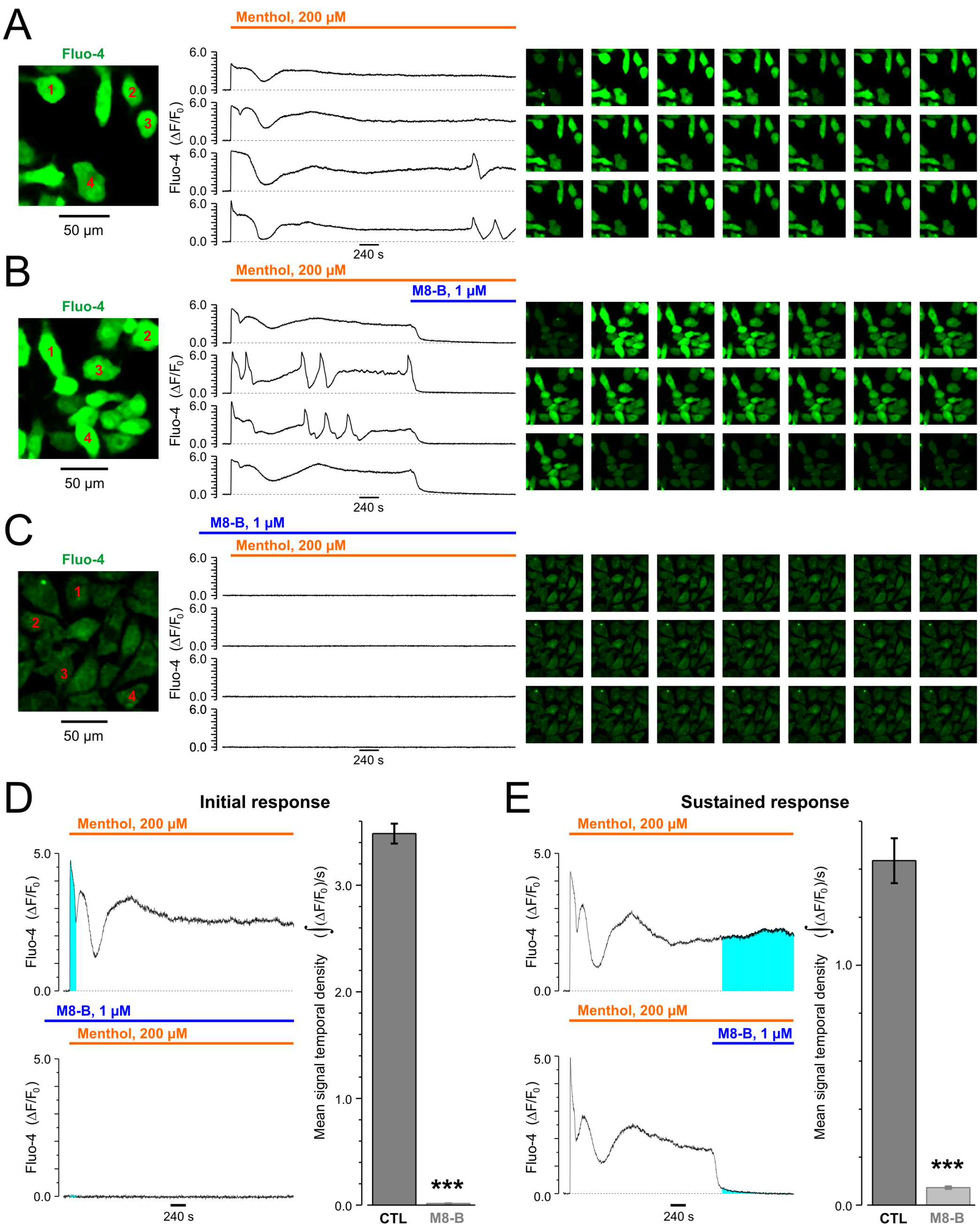

Supplemental Figure S1: Temporal profile of the $[Ca^{2+}]_c$ responses induced by 200 µM menthol in PC3 cells transfected with full-length TRPM8 (PC3-M8). Changes in $[Ca^{2+}]_c$, reported by confocal x-y time-series imaging (at 0.5 Hz) of fluo-4 fluorescence, were elicited by stimulation of TRPM8 with 200 µM menthol in PC3-M8 cells. Traces of the relative changes in fluo-4 fluorescence ($\Delta F/F_0$) in the cells depicted by the numbers (left) are shown from top to bottom, respectively (middle). The galleries (right) show (left to right, top to bottom) every 90th image captured during the imaging protocol. Note that initial $[Ca^{2+}]_c$ transient is followed by sustained $[Ca^{2+}]_c$ elevation persisting for at least 1 h (**A**). Also note that block of TRPM8 with selective inhibitor M8-B (1 µM) completely abolished both, the sustained response (**B-C**) and the initial $[Ca^{2+}]_c$ transient (**C**). The bar diagram plots (**D-E**, right) compare mean signal temporal densities, calculated as signal mass (left: $\int(\Delta F/F_0)$; cyan: periods of interest) per second for the initial (**D**) and the sustained (**E**) response in control (CTL; **D**: *n*= 197; **E**: *n*=65) and following TRPM8 inhibition (M8-B; **D**: *n*=85; **E**: *n*=132). ***P<0.001.

Supplemental S2

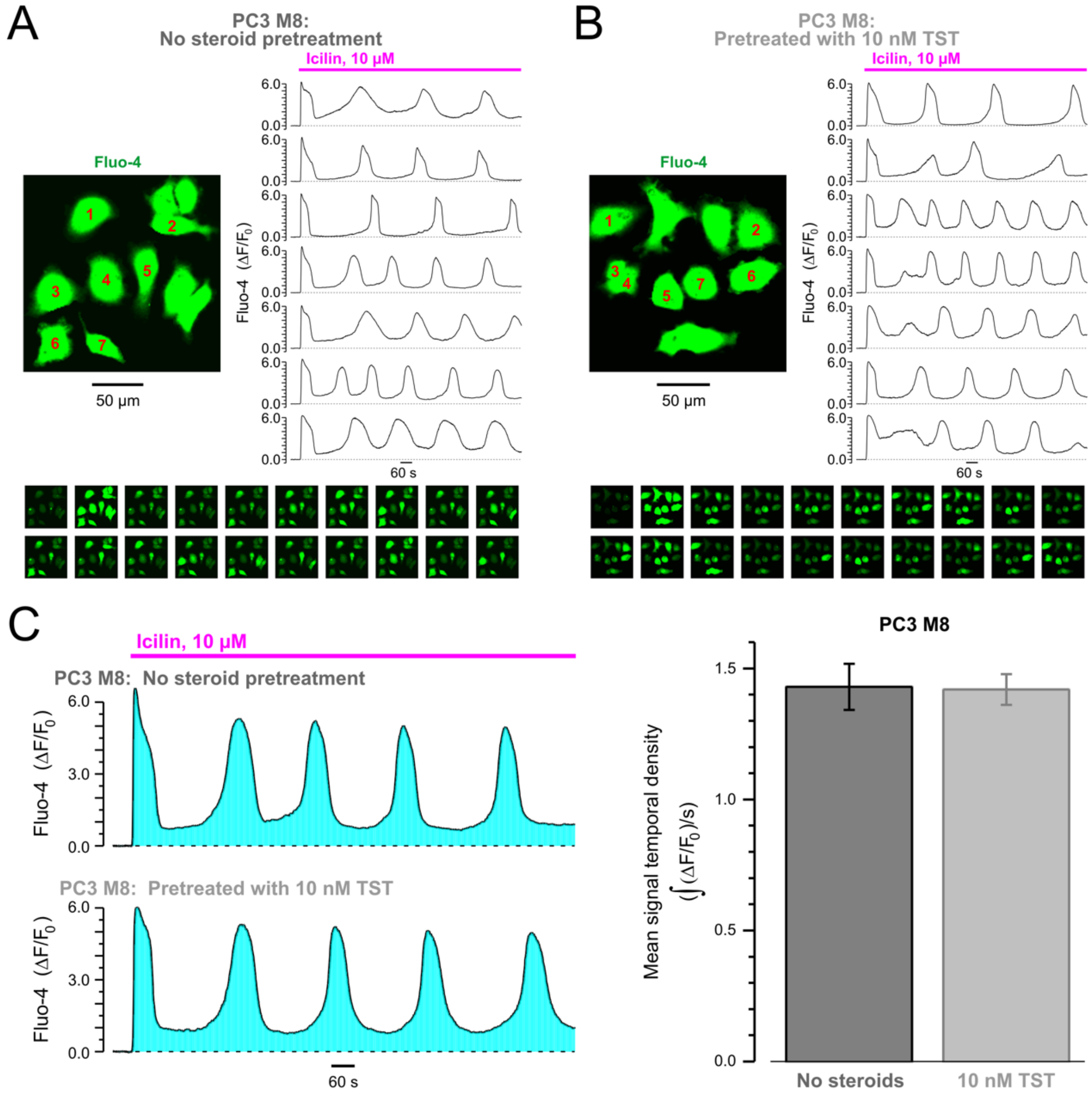

Supplemental Figure S2 : Icilin-induced $[Ca^{2+}]_c$ responses in PC3 cells transfected with full-length TRPM8 (PC3-M8) are insensitive to pretreatment with 10 nM testosterone (TST). Changes in cytosolic Ca^{2+} concentration ($[Ca^{2+}]_c$), reported by confocal time-series imaging (at 1 Hz) of fluo-4 fluorescence, were elicited by stimulation of TRPM8 with 10 μM icilin in PC3-M8 cells non-treated with steroids (**A**) or following 15-min incubation with 10 nM TST (**B**). The temporal profiles of the relative changes in fluo-4 fluorescence ($\Delta F/F_0$) in the cells depicted by the numbers (left) are shown from top to bottom, respectively (right). The galleries (bottom) show every 60th image captured during the *x-y* time series. (**C**) The bar diagram plot (right) compares corresponding mean signal temporal densities, calculated as signal mass (left: $\int(\Delta F/F_0)$) per second, in non-treated (*n*=49) and TST-treated (*n*=109) PC3-M8 cells. *P*= 0.92392.

Supplemental S3

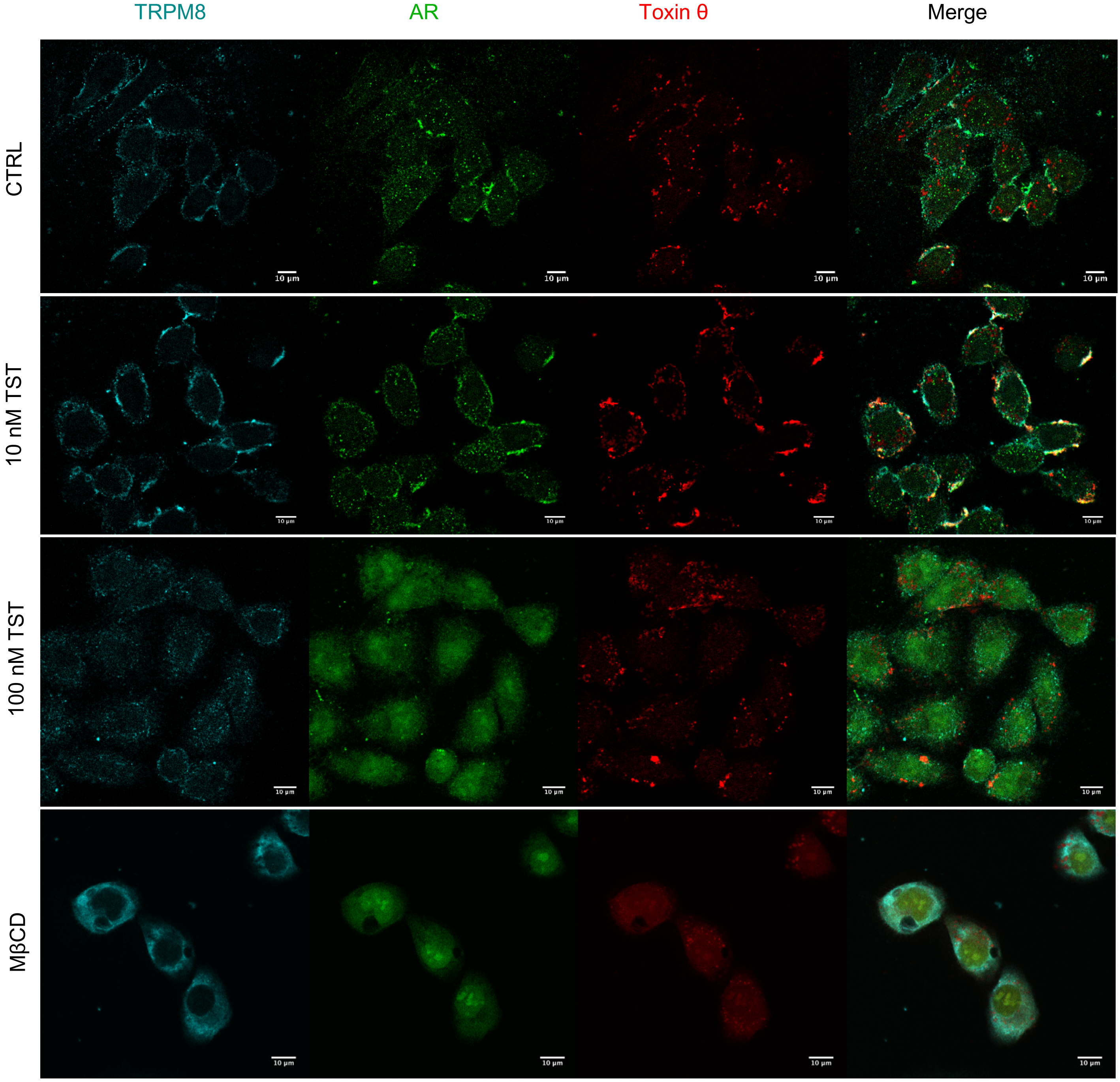

Supplemental Figure S3: Immunofluorescence was performed with LNCaP cells using TRPM8 and AR antibodies to label each protein and theta toxin-m-cherry to label cholesterol. LNCaP cells were treated with 10 or 100 nM TST 15 minutes before fixation. The cells were treated with 10 mM MßCD for 30 minutes at 37°C before fixation and used as negative control.

Supplemental S4

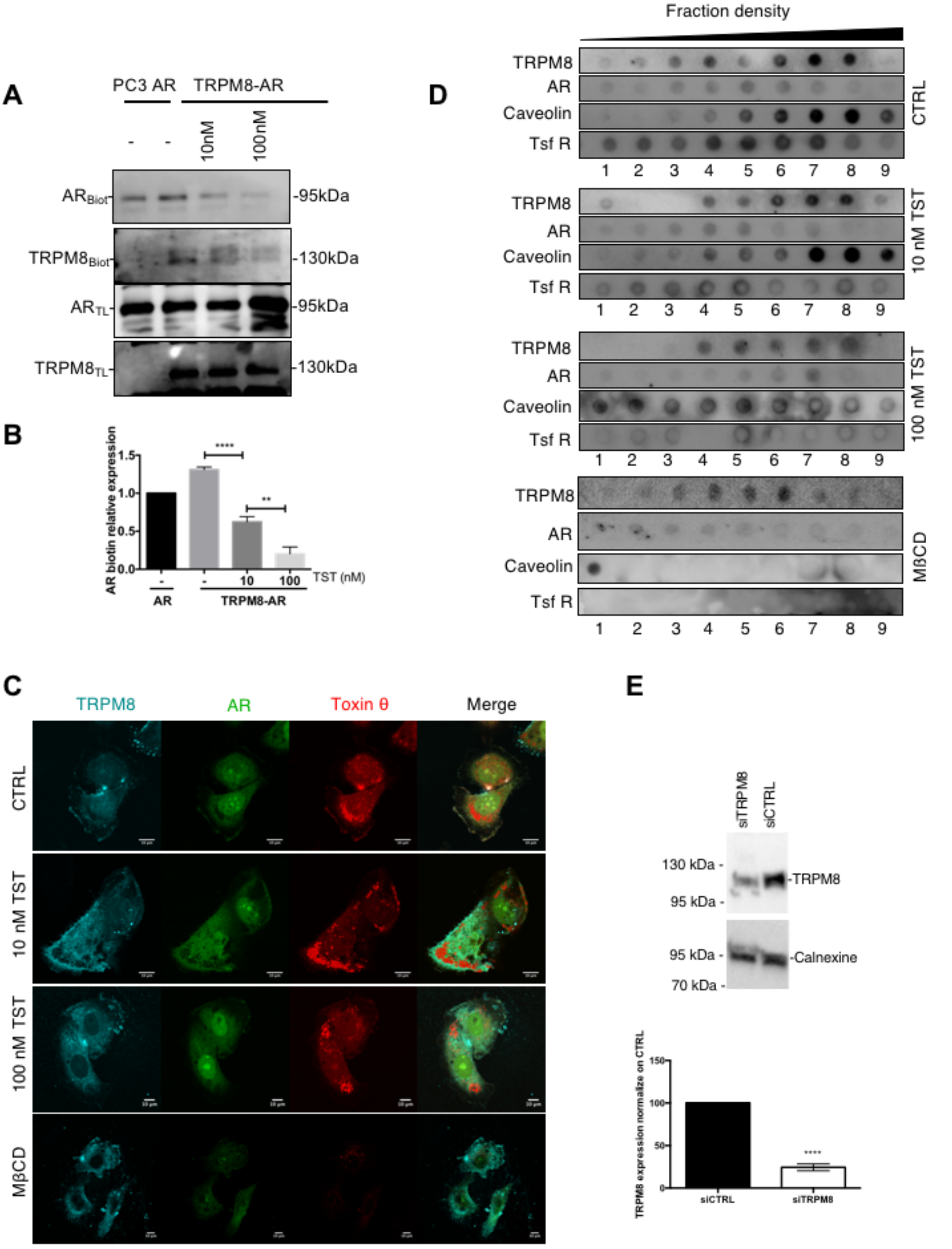

Supplemental Figure S4: TRPM8 and AR are found in the plasma membrane fraction of the heterologous system. (A) PC3 cells overexpressing TRPM8 and AR were treated with or without testosterone (10 or 100 nM) or vehicle (-) 15 minutes before incubation with 3 mg/ml

NHS-LC-LC biotin for 30 minutes at 4°C. Cells were lysed and incubated with neutravidin beads overnight and used for an immunoblot assay. The image in A represents the expression of TRPM8 and AR in the biotinylation fraction (Biot) and the total lysate (TL) and is representative of three independent experiments. Quantification of the AR expression in the plasma membrane fraction is presented in the bar graph **(B)**, ****=P<0.0001 (Student t-test). **(C)** Immunofluorescence was performed in PC3 cells overexpressing TRPM8 and AR using TRPM8 and AR antibodies to label each protein and theta toxin-m-cherry to label cholesterol. LNCaP cells were treated with 10 or 100 nM TST 15 minutes before fixation. The cells were treated with 10 mM MßCD for 30 minutes at 37°C before fixation and used as negative control. **(D)** Lipid rafts were extracted from PC3 cells that overexpressed TRPM8 and AR and were treated with 10 nM or 100 nM testosterone, vehicle (-) or 10 mM MßCD for 30 minutes at 37°C as negative control. Immunoreactivity against caveolin was used as a lipid raft marker, and immunoreactivity against transferrin receptor (Tsf R) was used as a negative control for lipid rafts. For each assay, N=3 independent experiments were performed. **(E)** Immune blot analysis showing TRPM8 expression in LNCaP cells treated with siControl or siTRPM8. siRNA against TRPM8 induced a a decrease by 73.75 ± 6.09 % of TRPM8 expression (N=3 independent experiments, **** = P ≤ 0.001, t-test).

Supplemental Movie S1: Changes in cytosolic Ca^{2+} concentration ($[Ca^{2+}]_c$), reported by confocal time-series imaging (at 1 Hz) of fluo-4 fluorescence, were elicited by stimulation of TRPM8 with 10 μM icilin in PC3-M8 cells non-treated with steroids.

Supplemental Movie S2: Changes in cytosolic Ca^{2+} concentration ($[Ca^{2+}]_c$), reported by confocal time-series imaging (at 1 Hz) of fluo-4 fluorescence, were elicited by stimulation of TRPM8 with 10 μM icilin in PC3-M8 cells following 15-min incubation with 10 nM TST.
